# High expression of neutrophil and monocyte CD64 with simultaneous lack of upregulation of adhesion receptors CD11b, CD162, CD15, CD65 on neutrophils in severe COVID-19

**DOI:** 10.1177/20499361211034065

**Published:** 2021-07-31

**Authors:** Malgorzata Karawajczyk, Lena Douhan Håkansson, Miklos Lipcsey, Michael Hultström, Karlis Pauksens, Robert Frithiof, Anders Larsson

**Affiliations:** Department of Medical Sciences, Clinical Chemistry, Uppsala University, Sjukhusvägen, entr 61, Uppsala, 751 05, Sweden; Department of Medical Sciences, Clinical Chemistry, Uppsala University, Uppsala, Sweden; Department of Surgical Sciences, Hedenstierna Laboratory, CIRRUS, Anesthesiology and Intensive Care, Uppsala University, Uppsala, Sweden; Department of Surgical Sciences, Anesthesia and Intensive Care Medicine, Uppsala University, Uppsala, Sweden; Department of Medical Cell Biology, Integrative Physiology, Uppsala University, Uppsala, Sweden; Department of Medical Science, Section of Infectious Diseases, University Hospital, Uppsala, Sweden; Department of Surgical Sciences, Anesthesia and Intensive Care Medicine, Uppsala University, Uppsala, Sweden; Department of Medical Sciences, Clinical Chemistry, Uppsala University, Uppsala, Sweden

**Keywords:** COVID-19, CD64, CD11b, CD15, CD65, CD 162, neutrophil, monocyte

## Abstract

**Background and Aims:**

The pronounced neutrophilia observed in patients with coronavirus disease 2019 (COVID-19) infections suggests a role for these leukocytes in the pathology of the disease. Monocyte and neutrophil expression of CD64 and CD11b have been reported as early biomarkers to detect infections. The aim of this study was to study the expression of receptors for IgG (CD64) and adhesion molecules (CD11b, CD15s, CD65, CD162, CD66b) on neutrophils and monocytes in patients with severe COVID-19 after admission to an intensive care unit (ICU).

**Methods:**

The expression of receptors was analyzed using flow cytometry. EDTA blood from 23 patients with confirmed COVID-19 infection was sampled within 48 h of admission to the ICU. Leukocytes were labeled with antibodies to CD11b, CD15s, CD65s, CD162, CD64, and CD66b. Expression of receptors was reported as mean fluorescence intensity (MFI) or the percentage of cells expressing receptors.

**Results:**

Results are presented as comparison of COVID-19 patients with the healthy group and the receptor expression as MFI. Neutrophil receptors CD64 (2.5 *versus* 0.5) and CD66b (44.5 *versus* 34) were increased and CD15 decreased (21.6 *versus* 28.3) when CD65 (6.6 *versus* 4.4), CD162 (21.3 *versus* 21.1) and CD11b (10.5 *versus* 12) were in the same range. Monocytes receptors CD64 (30.5 *versus* 16.6), CD11b (18.7 *versus* 9.8), and CD162 (38.6 *versus* 36.5) were increased and CD15 decreased (10.3 *versus* 17.9); CD65 were in the same range (2.3 *versus* 1.96).

**Conclusion:**

Monocytes and neutrophils are activated during severe COVID-19 infection as shown by strong upregulation of CD64. High monocyte and neutrophil CD64 can be an indicator of a severe form of COVID19. The adhesion molecules (CD11b, CD162, CD65, and CD15) are not upregulated on otherwise activated neutrophils, which might lead to relative impairment of tissue migration. Low adhesion profile of neutrophils suggests immune dysfunction of neutrophils. Monocytes maintain upregulation of some adhesion molecules (CD11b, CD162) suggesting the persistence of an increased ability to migrate into tissues, even during a severe stage of COVID-19. Future research should focus on CD64 and CD11b kinetics in the context of prognosis.

## Introduction

Viral infection often causes a rise in lymphocyte but not in neutrophil count in peripheral blood. In patients with coronavirus disease 2019 (COVID-19) an opposite trend has been observed, i.e., an elevated neutrophil count but not a lymphocyte increase. The progressing lymphocytopenia and neutrophilia are signs of a less favorable prognosis.^1,2^ Although neutrophils are potent antimicrobial cells, they can contribute to host tissue damage when hyperactive.^3^ For example, production of neutrophil extracellular traps (NET-osis) by activated neutrophils is a proposed contributing factor in organ damage in COVID-19 patients.^4^ Moreover, increased levels of neutrophil-associated cytokines originating from NLRP3 inflammasome is correlated with organ damage in COVID-19 patients.^5^ A pattern of 25 granulocyte-associated markers has been proposed recently to predict critical illness and mortality in COVID-19.^6^

Bacterial superinfections have been identified in 8% of hospitalized COVID-19 patients, but 72% of hospitalized COVID-19 patients have received antibiotics despite the lack of evidence for bacterial coinfection.^7^ Overuse of antibiotics results in increased antibiotic resistance.^8^ Antibiotic resistance results in longer hospital stay, high mortality and morbidity, and increased hospital burden.^9^ Generally, the risk of superinfection increases with hospitalization time and the length of time on a ventilator.^10^ Because inflammatory parameters are increased due to viral infection and organ damage, it might be difficult to distinguish the inflammation caused by COVID-19 from bacterial superinfections during hospitalization. This may be a reason for widespread antibiotic use in COVID-19 patients without verified bacterial infections.

The high-affinity receptor for immunoglobulin G (FcγRI) – CD (cluster of differentiation) 64 has an important role in innate immunity. FcγRI is a cyto-activating receptor: for example, its crosslinking induces cellular responses, including phagocytosis, the release of inflammatory mediators, antibody-dependent cell-mediated cytotoxicity, and antigen presentation.^11^ CD64 is expressed constitutively on monocytes, but in neutrophils it is stored inside the cell, and mobilized to the surface upon priming.^12^ Neutrophil expression of CD64 has been shown previously to perform well as a biomarker of bacterial infection,^13,14^ and even to distinguish bacterial from viral infections.^15,16^ Neutrophil CD64, as well as neutrophil and monocyte CD11b expression, has shown good discriminatory power between severe septic and non-septic intensive care unit (ICU) patients (operation, trauma, intoxication, cerebral hemorrhage) and in distinguishing infection from disease flare in patients with autoimmune disease.^17,18^ Neutrophil CD64 is a useful marker in the diagnosis and prediction of survival in ICU patients with ventilator-acquired pneumonia and even superior to procalcitonin and CRP in the diagnosis of sepsis in ICU patients.^19,20^

Leukocyte adhesion to endothelial cells and subsequent transmigration from the bloodstream to tissues is facilitated by the adhesion molecules: selectins and integrins and their ligands. CD15s, CD65s (E-selectin ligand), and CD162 (P-selectin ligand) facilitate rolling, and CD11b (a member of integrin family) promotes adhesion and thereby transmigration of leukocytes from the blood vessels to the extravascular compartment.^21^

The integrin receptor CD11b has been upregulated on neutrophils and monocytes upon exposure to viral and bacterial infections.^22^ It is suggested to be a useful marker of neonatal sepsis.^23^ A recent study of COVID-19 patients demonstrated a decrease in granulocyte CD11b expression.^6^ Blocking CD11b has been proposed to have a beneficial effect on sepsis outcome.^24^

Little is known about the expression of these molecules in infections. Inhibition of CD162 results in attenuation of leukocyte adhesion and extravasation to tissues in animal models of septic shock,^25^ which make selectin ligands attractive targets for clinical intervention. Neutrophil CD162 has been shown to decrease during exacerbation of COPD caused by infection.^26^ *In vitro*, interleukin–8(Il-8) and formyl-methionyl-leucyl-phenylalanine (f-MLP) downregulate CD15 and CD162.^27^

The knowledge of CD65s in the aspect of infection is scarce. *In vitro* experiments showed that the CD65 expression on neutrophils increases upon stimulation with f-MLP but not with IL-8.^27^ CD65 has been shown recently to play a role in the extravascular infiltration of acute myeloid leukemia cells.^28^

CD66b is a member of the carcinoembryonic antigen-related cell adhesion molecule 8 family.^29^ It is upregulated upon neutrophil activation due to mobilization of granules to the cell surface.^12^ CD66b is increased in rheumatic arthritis,^30^ bacterial and viral infection,^22^ and after surgery.^31^

Given the proposed role of neutrophils in COVID-19 infection, it seems likely that the neutrophil activation markers may be useful in monitoring inflammation and, by that, assess the outcome.

This study aims to investigate the activation of blood neutrophils and monocytes from COVID-19 patients by determining the expression of receptors for IgG (CD64), adhesion molecules (CD11b, CD162, CD65s, CD15s), and cell activation (CD66b), and evaluating whether they can be potential markers of inflammation in severe COVID19, especially in the context of hospital-acquired bacterial superinfection and to clinical prognosis assessment.

## Materials and methods

### Participants

All the patients were adults admitted to the general ICU at Uppsala University Hospital from 22 April 2020 to 17 May 2020. The patients were included after written informed consent was obtained from themselves or from their next-of-kin for patients unable to consent. COVID-19 was diagnosed by polymerase chain reaction for severe acute respiratory syndrome coronavirus 2 (SARS-Cov-2). The protocol of the study was registered *a priori* [ClinicalTrials.gov identifier: NCT04316884]. The study was approved by the National Ethical Review Agency (EPM; No. 2020–01623) and adheres to the ethical practices described in the Declaration of Helsinki and its subsequent revisions.

The control group comprised of 20 healthy blood donors who made their routine control complete blood count (CBC) in connection with their visit to the local blood center. Blood that was left over after performing the routine analyses was used for receptor expression measurements. Control samples were analyzed anonymously, according to the ethical permit from the Uppsala University Ethical Committee (Dnr: 01-367).

### Study design

EDTA blood for analysis of receptor expression were collected within 48 h after COVID-19 patients admission to the ICU. The control group consisted of health blood donors. Receptors expression was analyzed within 6 h from sampling.

Lithium heparin blood for C-reactive protein (CRP), procalcitonin, ferritin, N-terminal pro-brain natriuretic peptide (NT-pro-BNP), D-dimer, and troponin I was collected routinely at 0600 hours for all COVID-19 patients. For plasma markers, the normal reference ranges used at the Department of Clinical Chemistry and Pharmacology, Uppsala University Hospital, were used for comparison.

### Flow cytometry

#### Labelling of leukocytes with antibodies to cell surface antigens

Leukocytes in whole blood were fixed with 0.2% paraformaldehyde and the erythrocytes lysed by ammonium chloride according to Hamblin *et al*.^32^ After two washes with PBS containing 0.1% human albumin [PBS–0.1% human serum albumin (HSA)] the leukocytes were incubated for 30 min at +4°C in two tubes with fluorescence labelled antibodies. Tube 1 contained anti-CD11b-FITC [Beckman Coulter (BC) Marseilles, France], anti-CD14-PC7 (BC), anti-CD15s-BV421 (BD Biosciences, San Jose, CA, USA) and anti-CD66b-APC (BC), and tube 2 anti-CD65s-FITC (Bio-Rad, anti-CD14-PC7 (BC), Oxford, UK), anti-CD64-APC-AlexaFluor750 (BC), and anti-CD162-PE (BD Biosciences). After incubation, all the samples were washed twice with ice cold PBS and thereafter diluted in PBS–0.1% HSA. The tubes were placed in the dark at 4°C until the flow cytometry analysis was performed.

#### Flow cytometry analysis

Flow cytometry analysis was performed using the flow cytometer Navios (Beckman Coulter, Bromma, Sweden). Granulocytes were identified by their forward and side scatter dot-plot profile and monocytes by their expression of CD14. A gate was set around the granulocyte and the CD14 positive monocyte population. The proportion (%) of granulocytes and monocytes expressing the receptor and the mean fluorescence intensity (MFI) of the granulocyte and monocyte population were assayed. A minimum of 10,000 events in the granulocyte gate was analyzed.

### CRP, procalcitonin, ferritin, troponin I, NT-pro-BNP

Lithium heparinized plasma was used to analyze inflammation and organ damage markers. All analyses were performed on Architect ci16200 (Abbott, Abbott Park, IL, USA) as standard samples.

#### Statistical analysis

Data are presented as mean and standard error of mean (SEM). To describe the predictive value of the studied receptors for mortality after 30 days and their corresponding 95% confidence intervals (CI), area under the curve was obtained from the receiver operating characteristic curve (AUC-ROC) using MedCalc, version 14 (MedCalc Software, Ostend, Belgium). The best cut-off was defined at the maximal distance from ROC curve and the diagonal.

STATISTICA software, version 10 (StatSoft, Tulsa, OK, USA) was used for Spearman Rank and Mann–Whitney U calculations. Mann–Whitney *U* test was used for intergroup differences and *p* < 0.05 was considered significant throughout the study.

## Results

### Patient characteristics

Samples for flow cytometry were obtained from 23 patients (8 women, 15 men) with a median age of 64 (range 35–84) years. Patients were admitted to the ICU on the average of the 9th day of clinically defined disease (range 4–20 days). The main reason for admission was respiratory insufficiency. All patients had fever (median 39°C, range 38–40°C). The Simplified-Acute-Physiology-Score (SAPS 3) on arrival was 52 (range 32–69).^33^ The mean body mass index was 28 (range 21–43 kg/m^2^). The group for which flow cytometry samples were collected was not different from the full cohort of 53 COVID-19 patients treated at the ICU during this time. Table 1 shows the demographic details of the entire cohort and group for which neutrophil and monocyte receptors were measured. Dropouts from the whole cohort was due to the availability of the flow cytometry analysis.

**Table 1.** Patient characteristics: comparison of the whole cohort of patients in an ICU with the studied subgroup. The neutrophil marker group does not differ from the whole cohort.

	All ICU patients from 22/4 to 17/5		Neutrophil marker patients	
*n*	*n* = 53		*n* = 23	
Age (years, SD)	61	15	64	14
Female (*n*, %)	13	25%	8	35%
BMI (mean, SD)	30	6	29	6
SAPS3 (mean, SD)	52	8	53	10
Comorbidities				
Lung disease (*n*, %)	11	21%	4	17%
Hypertension (*n*, %)	24	45%	12	52%
Heart failure (*n*, %)	3	6%	3	13%
Ischemic heart disease (*n*, %)	6	11%	3	13%
Vascular disease (*n*, %)	8	15%	5	22%
Thromboembolic disease (*n*, %)	2	4%	2	9%
Liver failure (*n*, %)	1	2%	1	4%
Malignant disease (*n*, %)	0	0%	0	0%
Diabetes (*n*, %)	14	26%	6	26%
Neurological disease (*n*, %)	3	6%	2	9%
Psychiatric disease (*n*, %)	3	6%	1	4%
Complications/outcomes				
Thrombotic events (*n*, %)	8	15%	1	4%
Critical illness neuro/myopathy (*n*, %)	6	11%	3	13%
Delirium (*n*, %)	3	6%	2	11%
Secondary infection (*n*, %)	27	51%	9	39%
30-day mortality (*n*, %)	12	23%	6	26%
Medications				
Corticosteroid treatment (*n*, %)	4	8%	1	4%
ACEi/ARB treatment (*n*, %)	15	28%	10	43%
Anticoagulant treatment (*n*, %)	12	23%	6	26%

ACEi, angiotensin- converting enzyme inhibitor; ARB, angiotensin receptor blocker; BMI, body mass index; ICU, intensive care unit; SAPS3, Simplified Acute Physiology Score III; SD, standard deviation.

The control group for receptor measurements consisted of 20 healthy blood donors from the local blood collection center.

### Receptors

**Figure 1.**
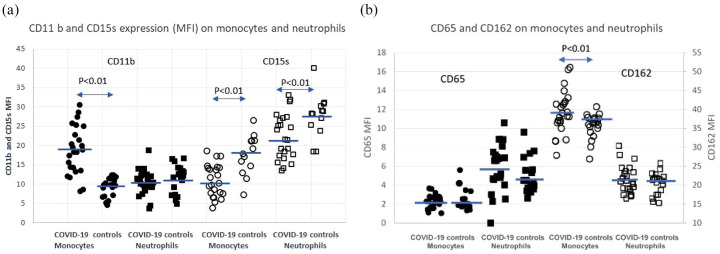
(a) Expression of CD11b and CD15s on monocytes and neutrophils. Bars represent median values. A difference is considered significant if *p* < 0.05 as calculated with the Benjamin–Hochberg test and *p* < 0.01 with the Mann–Whitney test. (•) CD11b monocytes, (■) CD11b neutrophils, (○) CD15s monocytes, (□) CD15s neutrophil. (b) Expression of CD65 and CD162 on monocytes and neutrophils. Bars represent the mean value. A difference is considered significant if *p* < 0.05 as calculated with the Benjamin–Hochberg test and *p* < 0.01 with the Mann–Whitney test. (•) CD65 monocytes, (■) CD65 neutrophils, (○) CD162 monocytes, (□) CD162 neutrophils. COVID-19, coronavirus disease 2019; MFI, mean fluorescence intensity.

Adhesion receptors: CD11b, CD15s, CD65s and CD162. Data are presented in Figure 1a,b and Table 2.

**Table 2.** Expression of receptors on neutrophils and monocytes in COVID-19 patients and healthy groups. Data are expressed as mean ± SEM and range (min–max).

Receptor expression per cell MFI	Patients Mean/±SEM/range	Healthy median/±SEM/range	*p* Value Mann–Whitney	Benjamin–Hochberg *p* value. Significant with f 0.05
CD11b Neutrophils	10.6 ± 0.64 (3.6–18.7)	11.7 ± 0.8 (4.8–16.6)	0.21	0.267350479*
CD11b Monocytes	19.1 ± 1.3 (8.25–30.6)	9.1 ± 0.6 (4.7–12.4)	0.000001*	0.000028*
CD66b Neutrophils	46.2 ± 3.1 (20.6–74)	34.02 ± 2.2 (15.9–55.7)	0.0136*	0.019776578*
CD15s Neutrophils	22 ± 1.3 (13.5–33.1)	27.7 ± 1.6 (18.5–40.1)	0.0135*	0.019776578*
CD15s Monocytes	11.1 ± 0.9 (3.8–18.6)	17.7 ± 1.4 (7.3–26.6)	0.0027*	0.004741654*
CD65 Neutrophils	5.9 ± 0.44 (2.3–10.6)	4.9 ± 0.44 (2.64–9.6)	0.0322*	0.043037243*
CD65 Monocytes	2.3 ± 0.14 (1.0–3.7)	2.32 ± 0.14 (1.32–5.6)	0.4114	0.414643309
CD 162 Neutrophils	21.6 ± 0.7 (16.5–30.4)	20.4 ± 0.64 (16.5–30.4)	0.3684	0.393062398
CD162 Monocytes	39.2 ± 1.2 (27.9–51.1)	36.7 ± 0.72 (26.9–40.7)	0.0018*	0.003659487*
CD64 Neutrophils	3.7 ± 0.6 (0.52–9.7)	0.67 ± 0.7 (0.39–1.7)	0.0000*	0.000001*
CD64 Monocytes	32.02 ± 2.1 (14.3–53.9)	16.3 ± 0.6 (8.99–21.4)	0.0000*	0.000033*
Proportion of cells expressing receptor %				
CD64 Neutrophils%	77.8 ± 5.6 (16.2–100)	16.3 ± 0.6 (6.9–55.9)	0.0000*	0.000001*

* Differences are considered significant if *f* < 0.05 as calculated with the Benjamin–Hochberg test and *p* < 0.01 the Mann–Whitney test.

COVID-19, coronavirus disease 2019; MFI, mean fluorescence intensity; SEM, standard error of the mean.

Expression of the integrin receptor CD11b increased significantly on monocytes but not on neutrophils in COVID-19 patients compared with healthy controls. Expression of CD15s decreased on both monocytes and neutrophils in the COVID-19 patient group compared with healthy controls. The level of the E-selectin ligand CD65s expression on monocytes and neutrophils did not differ between COVID-19 patients and healthy controls. The expression of the P-selectin ligand CD162 was slightly higher on monocytes from COVID-19 patients compared with healthy controls. Neutrophil CD162 expression showed similar levels in COVID-19 patients as in healthy controls.

#### High-affinity IgG receptor FcγRI CD64

**Figure 2.**
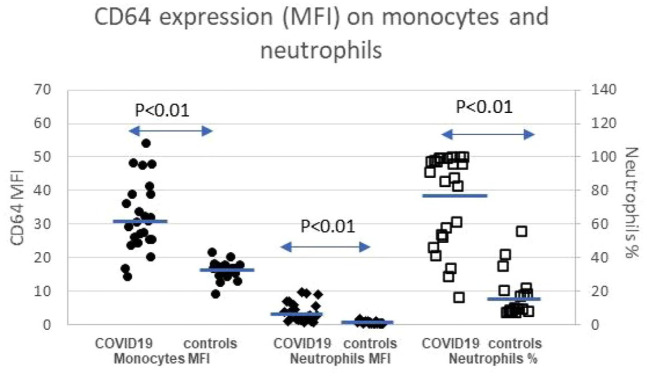
Expression of CD64 on monocytes and neutrophils. Bars represent the mean value. A difference is considered significant if *p* < 0.05 as calculated with the Benjamin–Hochberg test and *p* < 0.01 with the Mann–Whitney test. (•) CD64 monocytes MFI, (♦) CD64 neutrophils MFI, (□) CD64 neutrophils %. COVID-19, coronavirus disease 2019; MFI, mean fluorescence intensity.

Expression of CD64 on monocytes and neutrophils increased significantly in the COVID-19 patient group compared with the healthy controls. Likewise, a proportion of neutrophils expressing CD64 increased significantly (Figure 2 and Table 2).

#### Activation marker CD66b

The expression of CD66b on neutrophils was elevated significantly in COVID-19 patients compared with healthy controls. CD66b is not expressed on monocytes (Table 1).

### Inflammation markers CRP, ferritin, procalcitonin and organ dysfunction markers troponin I, NT-pro-BNP, and D-dimer

The mean plasma levels of the inflammation markers CRP, procalcitonin, and ferritin were elevated above the normal range in the COVID-19 patient group (Table 3). The CRP levels were above 50 mg/L in all but two of the COVID-19 patients. Procalcitonin levels above 0.25 μg/L were noted in 17/23 patients. Low procalcitonin levels <0.25 mg/L were seen in 7/23 COVID-19 patients. Plasma levels of NT-pro-BNP, troponin I and D-dimer were increased above the normal range in most of the patients (Table 3).

**Table 3.** Plasma levels of inflammatory and organ damage markers in COVID-19 patients.

Marker	Mean ± SEM	Min–max	Reference interval	Units
CRP	174 ± 19	14–351	<5	mg/l
Procalcitonin	0.67 ± 0.31	0.08–6.5	<0.05	μg/l
Ferritin	1613 ± 267	312–5563	25–310	μg/l
Troponin	16 ± 6.9	1–137	<16 women	ng/l
			<35 men	
NT-pro-BNP	425 ± 355	105–7330	Women <50 years <150	ng/l
			Women >50 years <330	
			Men <50 years <90	
			Men >50 years <230	
D-dimer	1.5 ± 0.15	0.2–3.1	Under 51 years <0.50	mg/l FEU
			51 years <0.51	
			51 < 0.52 etc.	
			90 years <0.9	

COVID-19, coronavirus disease 2019; CRP, C-reactive protein; FEU, fibrinogen equivalent units; SEM, standard error of the mean.

### Correlation between plasma inflammation markers and receptors

The correlations between plasma markers of inflammation and all measured receptors were calculated. Statistically significant correlations were found for CRP and procalcitonin (*p* = 0.0033), for neutrophil CD64% and procalcitonin (*p* = 0.00058) and for neutrophil CD64 MFI and procalcitonin (*p* = 0.018) (Table 4). There were no significant correlations between other receptors and CRP or procalcitonin (*p* < 0.05).

**Table 4.** Correlation between inflammation markers and expression of studied receptors calculated with Spearman rank. *p* < 0.05 was considered significant.

Marker	*N*	Spearman *R*	T(N-2)	*p* value
Procalcitonin and cd64neutrMFI	23	0.585	3.308	0.0033
Procalcitonin and cd64neutr%	23	0.663	4.058	0.000577
Procalcitonin and CRP	23	0.488	2.562	0.01816

CRP, C-reactive protein.

### Bacterial infection

Of the 23 patients, 20 (87%) received antibiotic therapy at sample collection. Only 10 had a microbiologically verified bacterial superinfection. Causative microorganisms were *Staphylococcus aureus*, *Haemophilus influenzae*, *Moraxella catarrhalis*, *Enterococcus faecalis*, *Escherichia coli*, *Serratia marcescens*, *Pseudomonas aeruginosa*, and *Candida albicans*; 7/10 had polymicrobial flora. Microorganisms were present in different biological materials such as blood, urine, tracheal secretion, or nasopharyngeal swabs. There was no difference in receptor levels, procalcitonin, or CRP between patients with positive and negative bacterial culture.

### Prognostic values of the studied receptors and plasma markers

**Figure 3.**
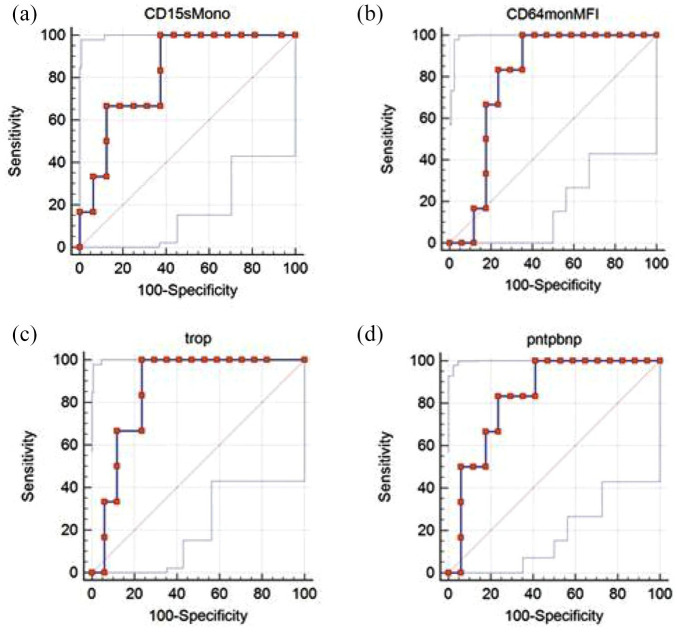
ROC curves for predicting death. Dotted lines show ROC curve, light grey CI (a) monocyte CD15 AUC = 0.82 (b) monocyte CD64 AUC = 0.79, (c) troponin AUC = 0.86 and (d) NT-pro-BNP AUC = 0.833. AUC, area under the ROC curve; CI, confidence interval; COVID-19, coronavirus disease 2019; MFI, mean fluorescence intensity; ROC, receiver operating characteristic.

Of the 23 COVID-19 patients, 6 died within 30 days from admission to hospital. To analyze the ability of receptors, inflammation, and organ damage markers to distinguish between survivor and non-survivor in the whole COVID-19 group, we calculated the ROC curve for each of the parameters. Data are presented in Table 5 and Figure 3.

**Table 5.** The diagnostic performance of studied receptors expression and plasma levels of cardiac damage markers.

Marker	AUC	SE	95% CI	Significance level *p* (area = 0.5)	Cut-off value	Sensitivity	Specificity	NPV	PPV
mCD15s	0.823	0.09	0.603–0.951	0.0004	⩽11.7	100	62.5	100	50
mCD64	0.794	0.093	0.576–0.932	0.0015	>29.1	100	64.7	100	50
NT-pro-BNP	0.833	0.087	0.621–0.954	0.0001	>595	83.3	76.5	93	56
Troponin I	0.863	0.077	0.656–0.969	<0.0001	>19	100	76.4	100	50

AUC, area under the curve; CI, confidence interval; NPV, negative predictive value; PPV, positive predictive value; SE, standard error.

Among receptors, the strongest association with mortality was shown by monocyte CD15 (*p* = 0.0004), followed by monocyte CD64 (*p* = 0.0015). Other receptors did not reach a statistical significance of *p* < 0.05. Among the organ damage markers, NT-pro-BNP (*p* = 0.0001) and troponin I (*p* < 0.0001) were also significantly associated with mortality. All four markers showed strong negative predictive value at chosen cut-off points. None of the clinically used inflammation markers (CRP, procalcitonin and ferritin) were associated significantly with mortality.

## Discussion

In some patients, COVID-19 can take a very severe course, with pronounced inflammation leading to severe tissue damage. Neutrophils and monocytes play an important role in the development of exaggerated inflammatory response. Expression of surface receptors on neutrophils and monocytes reflects state of their activation and can be used as a marker of ongoing inflammation.

The main result of this study is that neutrophils and monocytes are activated in patients with critical COVID-19 infections as shown by upregulation of CD64. No upregulation of neutrophil adhesion molecules (CD11b, CD15s, CD65s, CD162) was observed, whereas monocytes showed upregulation of adhesion molecules (CD11b and CD162).

### Receptor expression

Selectins are responsible for the first step of adhesion to the blood vessel wall, e.g., rolling and integrins facilitates the second step, e.g., adherence. Neutrophils and monocytes express ligands for the P- and E-selectin (CD162, CD65, CD15s) and integrin receptor (CD11b).

Our group of COVID-19 patients showed a strong upregulation of the beta2-integrins (CD11b) on monocytes but not on the neutrophils, which differs from reports on influenza and bacterial infections but aligns with the report on COVID-19.^6,22^

Severe COVID-19 infection did not significantly change neutrophil expression of the selectin ligands CD162 and CD65s, whereas there was a reduction in expression of CD15s.

Lack of upregulation of the neutrophil selectin ligands (CD162 and CD65s) and integrin receptor (CD11b) may impair extravascular neutrophil transmigration to tissues and lead to an increased number of neutrophils in peripheral blood. Neutrophilia is characteristic of severe COVID-19 and can be due to increased bone marrow production, decreased migration from blood to tissues, or both. Impaired neutrophil extravascular migration can be a part of immune paralysis like that observed in sepsis patients or just part of a regulatory mechanism restricting the number of neutrophils in the tissue. The patients in our study were sampled, on average, on the 11th day of disease, making it impossible to assess whether normal receptor levels reflect a lack of activation or emerging inhibition due to regulatory mechanisms. The differences between previous studies and ours may also be caused partly by the kinetics of the CD11b expression. CD11b has been shown to have rapid up- and down-regulation depending on the activator.^17,34,35^ Lack of upregulation of neutrophil CD11b, CD162, CD15s, and CD65s in otherwise activated neutrophils can be a sign of immune dysfunction in COVID-19.

Pronounced increase of monocyte CD11b together with slight increase of monocyte CD162 may be enough to sustain enhanced monocyte migration even in the late stage of disease.

CD64, the high-affinity receptor for IgG, is expressed constitutively on monocytes but neutrophils express CD64 upon priming.

In the COVID-19 patient group, CD64 was strongly upregulated on neutrophils and monocytes compared with healthy controls. Similar findings were reported in children with multisystem inflammatory syndrome associated with SARS-CoV-2 infection and adults with severe COVID-19.^6,36^ Very high neutrophil CD64 (MFI > 1.8 and >80%) has been seen in patients with bacterial infection.^22^ Lower-grade CD64 expression has been observed in patients with influenza or trauma.^16,17^

The question arises whether the very high expression of neutrophil and monocyte CD64 in COVID-19 patients is due to neutrophil activation by bacterial superinfection or by inflammation caused by COVID-19. In the first case scenario, CD64 could be used as a marker for upcoming bacterial superinfection; in the second case, it could be a prognostic marker for tissue damage by inflammation. Our results do not conclusively support the hypothesis about the relation between bacterial superinfection and neutrophil CD64. A reason could be that most of the patients received broad spectrum antibiotics, which protects from infection. Another reason could be that inflammation in severe COVID-19 causes high upregulation of CD64. Kinetic studies of CD11b and CD64 before the start of antibiotic therapy are needed to clarify their usefulness as markers of bacterial superinfection.

High upregulation of both neutrophil and monocyte CD64 seems be a characteristic feature of severe COVID-19. An increase in neutrophil CD66 in another sign of neutrophil activation.

### Correlation between inflammation markers and receptors

The relation between CRP and procalcitonin was significant but quite weak. A large variation in CRP levels, procalcitonin, and ferritin observed in the patient group reflects complex regulatory mechanism of immune response in each individual patient. The correlation between neutrophil CD64 and procalcitonin was a little bit stronger than that between CRP and procalcitonin, suggesting that neutrophil CD64 and procalcitonin increase are partially regulated by similar mechanisms. Both markers have been proposed previously by many as specific markers of bacterial infection.^13,23,37^ The lack of correlation between a clinically used biomarker of infection such as CRP and procalcitonin and monocytes receptors such as monocyte CD11b, and monocyte CD64 MFI, suggest different regulatory mechanisms for each marker. This makes monocyte receptors interesting for future studies as an independent marker of inflammation in COVID-19.

### Prognostic values of markers

The cardiac damage markers in COVID-19 group showed associations with mortality according to the ROC analysis. This is in agreement with other reports on COVID-19.^38^ The predictor value of the cardiac damage parameters is related to cardiovascular insufficiency in critical illness. Plasma inflammation markers did not reach significant associations with death in our study group. This is contrary to the finding of many others, but there was significant heterogeneity between studies.^39^

Among cell receptors, even a slight depression of monocyte CD15s seems to indicate a risk for death. This is a new finding. The role of CD15s in infections is not very well known but increased numbers of CD15s positive neutrophils has been observed in neonatal sepsis. *In vitro*, incubation with Il-8 and f-MLP has been shown to cause a decrease in CD15 expression. Monocyte CD64 was highly upregulated in COVID-19 patients, and this increase has also a predictive value for a poor outcome. This has not been reported before. The bearing of monocyte markers on mortality can be related to the ability of activated monocytes for cytokine production and regulating T-cells, as increased monocyte CD64 implies monocyte activation and cytokine production.^40,41^ On the other hand, the lack of relationship between neutrophil activation expressed as increase CD64 and mortality in our group was unexpected, as activation of neutrophils has been shown to predict critical illness and mortality in COVID-19.^42^ Neutrophil activation has also correlated with organ damage,^4,5,43^ and increased granulocytic CD64 has been shown to discriminate well between mild and severe forms of COVID-19.^6^

### Limitation of the study

Small number of patients and short time of observation are limiting factors of the study.

## Conclusion

Neutrophils and monocytes are activated differently during severe COVID-19. Both have highly upregulated CD64. We propose that high neutrophil and monocyte CD64 can be used as a hallmark of severe COVID-19.

Adhesion molecules (CD11b, CD162, CD65 and CD15) are not upregulated on otherwise activated neutrophils, which can lead to relative impairment of tissue migration. Low adhesion profile of neutrophils suggests immune dysfunction. Monocytes maintained upregulation of some adhesion molecules (CD11b, CD162) suggesting the persistent increased ability to migrate into tissues, even during a severe stage of COVID-19. Future kinetic studies should examine whether neutrophil CD64 and CD11b expression, alone or in combination with other receptors, can be used as a predictive marker of prognosis in COVID-19.
